# Long-distance aerial dispersal modelling of *Culicoides* biting midges: case studies of incursions into Australia

**DOI:** 10.1186/1746-6148-10-135

**Published:** 2014-06-19

**Authors:** Debbie Eagles, Lorna Melville, Richard Weir, Steven Davis, Glenn Bellis, Myron P Zalucki, Peter J Walker, Peter A Durr

**Affiliations:** 1 CSIRO Animal, Food and Health Sciences, 5 Portarlington Rd, 3220 Geelong, Victoria, Australia; 2 School of Biological Sciences, The University of Queensland, 4072 Brisbane, Queensland, Australia; 3 Department of Primary Industry and Fisheries, Berrimah Veterinary Laboratories, GPO Box 3000, 0801 Darwin, Northern Territory, Australia; 4 Department of Agriculture, Northern Australia Quarantine Strategy, PO Box 37846, 0821 Winnellie, Northern Territory, Australia

**Keywords:** Culicoides, Bluetongue, Atmospheric dispersal modelling, Surveillance

## Abstract

**Background:**

Previous studies investigating long-distance, wind-borne dispersal of *Culicoides* have utilised outbreaks of clinical disease (passive surveillance) to assess the relationship between incursion and dispersal event. In this study, species of exotic *Culicoides* and isolates of novel bluetongue viruses, collected as part of an active arbovirus surveillance program, were used for the first time to assess dispersal into an endemic region.

**Results:**

A plausible dispersal event was determined for five of the six cases examined. These include exotic *Culicoides* specimens for which a possible dispersal event was identified within the range of two days – three weeks prior to their collection and novel bluetongue viruses for which a dispersal event was identified between one week and two months prior to their detection in cattle. The source location varied, but ranged from Lombok, in eastern Indonesia, to Timor-Leste and southern Papua New Guinea.

**Conclusions:**

Where bluetongue virus is endemic, the concurrent use of an atmospheric dispersal model alongside existing arbovirus and *Culicoides* surveillance may help guide the strategic use of limited surveillance resources as well as contribute to continued model validation and refinement. Further, the value of active surveillance systems in evaluating models for long-distance dispersal is highlighted, particularly in endemic regions where knowledge of background virus and vector status is beneficial.

## Background

*Culicoides* (Diptera: Ceratopogonidae) are small, haematophagous insects present on all continents except Antarctica. Whilst their movements for host seeking, feeding and breeding are usually over distances of less than 2 km [1], they can be dispersed up to hundreds of kilometres by the wind [2]. This so-called long-distance dispersal is particularly important because *Culicoides* are vectors for a range of pathogens, including viruses, bacteria and protozoa, some of which cause disease in humans or animals [3]. It is for this reason that their long-distance movements have been extensively modelled, particularly with respect to transmission of livestock diseases.

One such disease of ruminant livestock is bluetongue disease (BT), caused by the arbovirus of the same name. Bluetongue virus (BTV), for which certain *Culicoides* species are the only confirmed vectors, is endemic in many tropical and sub-tropical regions of the world. Whilst disease outbreaks in these regions are rare, infection of susceptible ruminants in non-endemic regions often results in clinical disease, particularly in sheep [4]. Modelling of the long-distance dispersal of *Culicoides* vectors has predominantly focussed on such outbreaks of disease, in cases where dispersal of infected vectors is considered the most plausible route of virus transmission [5-8]. Recently, the pathways of dispersal of *Culicoides* into northern Australia, from eastern Indonesia, Timor-Leste and Papua New Guinea (PNG) have been modelled [9,10]. Eagles *et al*.’s [10] analysis differed from previous analyses of long-distance *Culicoides* movement, in that retrospective assessment of a 15 year period was used to establish the likely spatio-temporal patterns of their dispersal within a BTV-endemic region.

Whilst aerial collections [11-14] have provided some evidence supporting the concept of long-distance dispersal of *Culicoides*, confirmation of their exact pathways of windborne movement is not currently possible [6]. For larger insects such as moths, migration pathways can be directly monitored using entomological radars [15,16] and, where applicable, these results can be used to validate predictive models [17]. Such radars are not yet sensitive enough to detect and distinguish *Culicoides*, which measure 1–3 mm in length [18]. Artificial tagging and recapturing can also be used to directly assess dispersal events [19]. Whilst *Culicoides* have been successfully tagged and recaptured, this has been attempted only for short vegetative flights, and even in these studies the recapture rates were very low (<0.5-2.1%) [1,20]. The primary evidence for *Culicoides* dispersal has been outbreaks of *Culicoides*-borne disease in non-endemic regions. In these cases, the date of onset of clinical signs, the implausibility of other means of virus introduction and the BTV status in possible source regions have been used, together with model outputs to build a case for the most likely dispersal pathways. More recently, genetic data have been used as evidence for the movement of haematophagous insects across long distances. Chapman *et al.*[21] demonstrated gene flow between mosquito populations from PNG and northern Australia, indicating the frequent movement of mosquitoes across the Torres Strait. Although genetic tools have yet to be explored for populations of Australian species of *Culicoides*, phylogeographical analyses have been used to demonstrate multiple incursions of *C. imicola* Kieffer into the northern Mediterranean basin from northern Africa [22].

In a BTV-endemic region, in the absence of clinical disease, the concept of evaluating predicted dispersal pathways is seemingly more difficult. However, by utilising the results of routine surveillance an independent assessment of the model is possible. In Australia, BTV is endemic in the north, where it does not currently cause clinical disease [23]. Despite this, the threat of incursion of a more pathogenic BTV, in addition to the ongoing requirement for monitoring to inform the implementation of live animal export protocols, necessitates regular, Australia-wide surveillance for BTV and its *Culicoides* vectors [24,25]. One specific objective of this surveillance is to provide early warning of incursions of exotic BTVs and *Culicoides* into the north [26]. In 2007, the first novel serotype detected in Australia for over 20 years, BTV-7, was isolated from sentinel cattle in the Northern Territory [27]. BTV-2 was detected for the first time the following year, in the same location [28]. Between 2009 and 2012, single specimens of exotic *Culicoides* species were collected in traps across northern Australia on four occasions (Bellis *et al*., in prep).

Here we use all cases of novel BTV and exotic *Culicoides* detections in northern Australia since 2007 to evaluate the model of *Culicoides* movement established in the previous studies [10]. These incursions, and their associated pathways of dispersal, can be considered as supportive of the previous model’s results. However, we also propose that such models cannot be fully ‘validated’ on surveillance (either active or passive) alone, but that surveillance and dispersal models can be used together to indicate likely patterns and pathways of dispersal, and to highlight areas requiring further research and surveillance.

## Methods

### The collections

**Figure 1:**
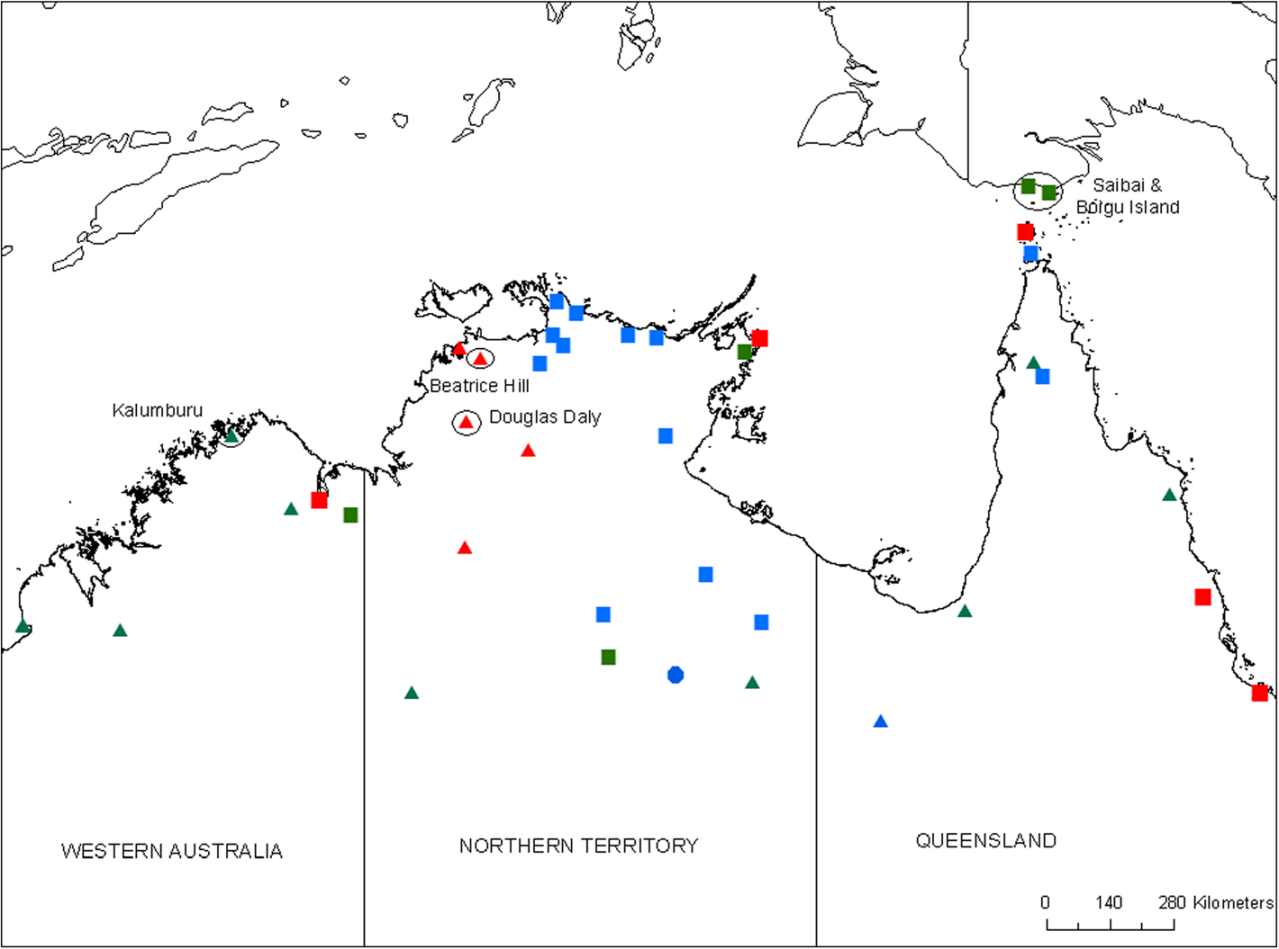
**Map of northern Australia showing all sentinel herd and vector collection sites used during the evaluation period (2007–2012).** Symbol shape represents the collections conducted at that site (circle – sentinel herd only, square – entomology only, triangle – both), colour represents the frequency of collections (red – regular (10-12/year), green – irregular (5-9/year), blue – rarely (4/year). Sites at which novel virus or exotic *Culicoides* have been collected, as listed in Table 1, are named and circled.

All of the exotic *Culicoides* were identified in routine collections conducted by the National Arbovirus Monitoring Program (NAMP), except the two detections in the Torres Strait that were detected through the Australian Department of Agriculture’s Northern Australia Quarantine Strategy (NAQS) program (Figure 1, Table 1) [29]. These collections make use of green LED light traps [30], generally set for 2–3 consecutive nights. All specimens are identified to species level [31] based on morphological characteristics.

**Table 1:** **Collection dates, dispersal windows and likely dispersal dates (see text for details) for each of the *Culicoides *** **and BTV isolates**

**Species/Isolate**	**Collection date (site)**	**Previous collection at this site**	**Hypothesised dispersal window**	**Plausible dispersal dates**
*C. nudipalpis*	22-24 Mar 2012 (Kalumburu)	22-24 Feb 2012	1-24 Mar	14-16 Mar
*C. flavipunctatus*	4-8 Nov 2010 (Saibai Is)	5-8 Oct 2012	14 Oct – 8 Nov	15-17 Oct
*C. palpifer, C. flavipunctatus*	5 May 2009 (Douglas Daly)	7-8 Apr 2009	14 April – 5 May	None
*C. orientalis*	24-27 Mar 2009 (Boigu Is)	23-26 Feb 2009	3 - 27 Mar	3-13 Mar, 17–22 Mar
BTV-2	10 Jan 2008 (Beatrice Hill)	3 Jan 2008	1 Dec 07–8 Jan	3-4 Jan
BTV-7	15 Mar 2007 (Beatrice Hill)	8 Mar 2007	1 Dec 06–13 Mar	14-16 Jan, 3–5 Mar

Each of the novel BTVs were identified in routine collections from a NAMP sentinel herd at Beatrice Hill, approximately 50 km SE of Darwin (Figure 1). Blood was collected from each of the cattle for virus isolation on a weekly basis and serology on the same cattle was conducted monthly [27]. Sampling of animals was conducted under Charles Darwin University Animal Ethics Committee approval, Project A11033. Virus isolation was conducted in two parallel systems [32].Collections of both vectors and viruses were made at a number of other NAMP sites in northern Western Australia (WA), Queensland (QLD) and the Northern Territory (NT) during the period in which these incursions took place (2007–2012) (Figure 1).

### Dispersal windows

A hypothesised ‘dispersal window’ during which the incursion event was most likely to have occurred was determined for each detection (Table 1). For the exotic *Culicoides* collected, the duration of the dispersal window equated to the maximum lifespan of an adult insect, based on the assumption that the collected specimen itself was dispersed and collected. The length of the adult lifespan was set at 21 days, based on previous studies [18].

The dispersal window for the novel BTVs was set as all dates between the previous December up to two days prior to the collection date. The latter is to allow for the minimum period for detection of virus in the blood, which coincides with the incubation period (time between infection and onset of clinical signs, where present). The incubation period is host species and virus dependent, ranging from 2–8 days [33]. December was chosen as the start of the dispersal window as, based on previous studies, this appears to be the start of the seasonal incursion risk period [10].

### The model

Within the dispersal windows likely dates of incursion were explored utilising the Hybrid Single Particle Lagrangian Integrated Trajectory Model (HYSPLIT) (Version 4), an atmospheric dispersion model (ADM). This model has previously been described [10] and elsewhere in more technical detail [34]. The model was used in particle mode for dispersion, which allows for transport of particles with mean wind and a random component to account for turbulence. The maximum allowable altitude was 1000 m, meaning that *Culicoides* particles transported beyond this height were no longer available to the model. Both ‘dry’ (gravitational) and ‘wet’ (rainfall) deposition were permitted.

**Figure 2:**
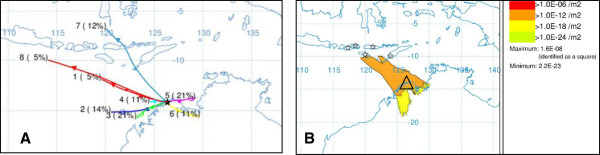
**Method of assessment of dispersal pathways. A**. Clustered back-trajectories from the incursion site. **B**. Source region defined and putative source sites selected (stars). Dispersal from the source sites at dusk and dawn assessed. Dispersal to Kalumburu (triangle) on 16 March 2012 is shown in this figure. The relative numbers of dispersed *Culicoides* are represented by the colours in the dispersal figure.

Twenty hour back-trajectories were generated at 6-hourly intervals for the period under assessment (Figure 2). The clustering component of the HYSPLIT model was used to aggregate like-trajectories, as described in Eagles *et al*. [10]. Based on the direction and distance of the clustered trajectories, the potential source area was defined for each incursion and within this area, putative point source locations were specified. In the model *Culicoides* were ‘released’ from each of the putative source sites, at dusk and dawn on every date on which a dispersal event appeared possible, based on the back-trajectory results. A total of 1000 particles (representing *Culicoides*) were ‘released’ over a 3 hour period. The flight/dispersal time was set at 20 hours, based on previous studies which suggest dispersal up to this period may be possible [2]. Figures were generated in ArcGIS Desktop 10.1.

### Meteorological input

The meteorological dataset used as input for the model was the Global Data Assimilation System (GDAS), available at three hour intervals with a global resolution of 1 degree latitude/longitude (approximately 100 km^2^) [35]. The GDAS data is considered suitable for determining pathways of dispersal over long distances.

## Results

**Figure 3:**
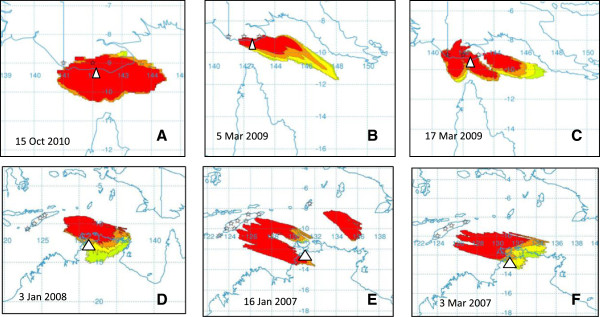
**Representative dispersal events for *C. flavipunctatus *collected on Saibai Island (A), *C. orientalis *collected on Boigu Island (B, C), BTV-2 collected at Beatrice Hill (D), BTV-7 (E, F) collected at Beatrice Hill.** The stars represent the putative source sites and the white triangle represents the collection site. The relative numbers of dispersed *Culicoides* are represented by the colours in the dispersal figure, with red the greatest number, followed by orange, yellow and light green.

An associated dispersal period and source region was determined for five of the six investigated incursions (Table 1, Figures 2, 3). Below we present the findings for each incursion.

### Case 1: *C. nudipalpis* Delfinado, Kalumburu, 22–24 March 2012

Collections were made on the nights 22–24 March 2012 at a site near Kalumburu, on the northern coastline of WA [23]. The previous collection at this site was made on 22–24 February 2012. Almost 8000 *Culicoides* were collected in March (compared to fewer than 1000 in February), and all were identified to species level. *Culicoides austropalpalis* Lee & Reye was the most abundant species, with large numbers of *C. bundyensis* Lee & Reye and *C. brevitarsis* Kieffer (all established species) also collected. A single specimen was morphologically identified as the exotic species *C. nudipalpis*. Subsequent collections at Kalumburu did not yield further specimens of this species, although almost 4000 *Culicoides* were collected and identified in the following six months (Luke Halling, Senior Entomologist, NAQS, pers comm.).

The dispersal window was defined as 1–24 March and seven putative source sites were assessed, ranging from Denpasar, Bali in the west to West Timor in the east. From 14–16 March dispersal was possible to Kalumburu from as far west as Lombok and as far east as West Timor (Figure 2). *Culicoides nudipalpis* is known from all of these islands [36,37]. Whilst molecular analysis of Indonesian specimens of *C. nudipalpis* has not been possible, the WA specimen has been shown to be 99.6% similar to *C. nudipalpis* from neighbouring Timor-Leste, based on analysis of the cytochrome oxidase I (COI) gene (Bellis *et al*., in prep).

### Case 2: *C. flavipunctatus* Kitaoka, Saibai Island, 4–8 November 2010

A single female specimen of *C. flavipunctatus* was collected on Saibai Island in the Torres Strait in November 2010 [38]. The previous collection at this site was on 5–8 October 2010. At its northern most point, Saibai Island is less than 4 km from the PNG mainland. Despite this short distance, vegetative movements over an expanse of water would seem unlikely, although transport on vessels cannot be categorically ruled out.The dispersal window was set at 15 October – 8 November. Based on 20 hour back-trajectories, dispersal is most likely to have occurred in the period 15–17 October, from southern PNG. Dispersal between the PNG mainland and Saibai Island may have occurred in less than one hour on 15 October 2010 (Figure 3A).

### Case 3: *C. palpifer* Das Gupta & Ghosh and *C. flavipunctatus*, Douglas Daly, 5 May 2009

**Figure 4:**
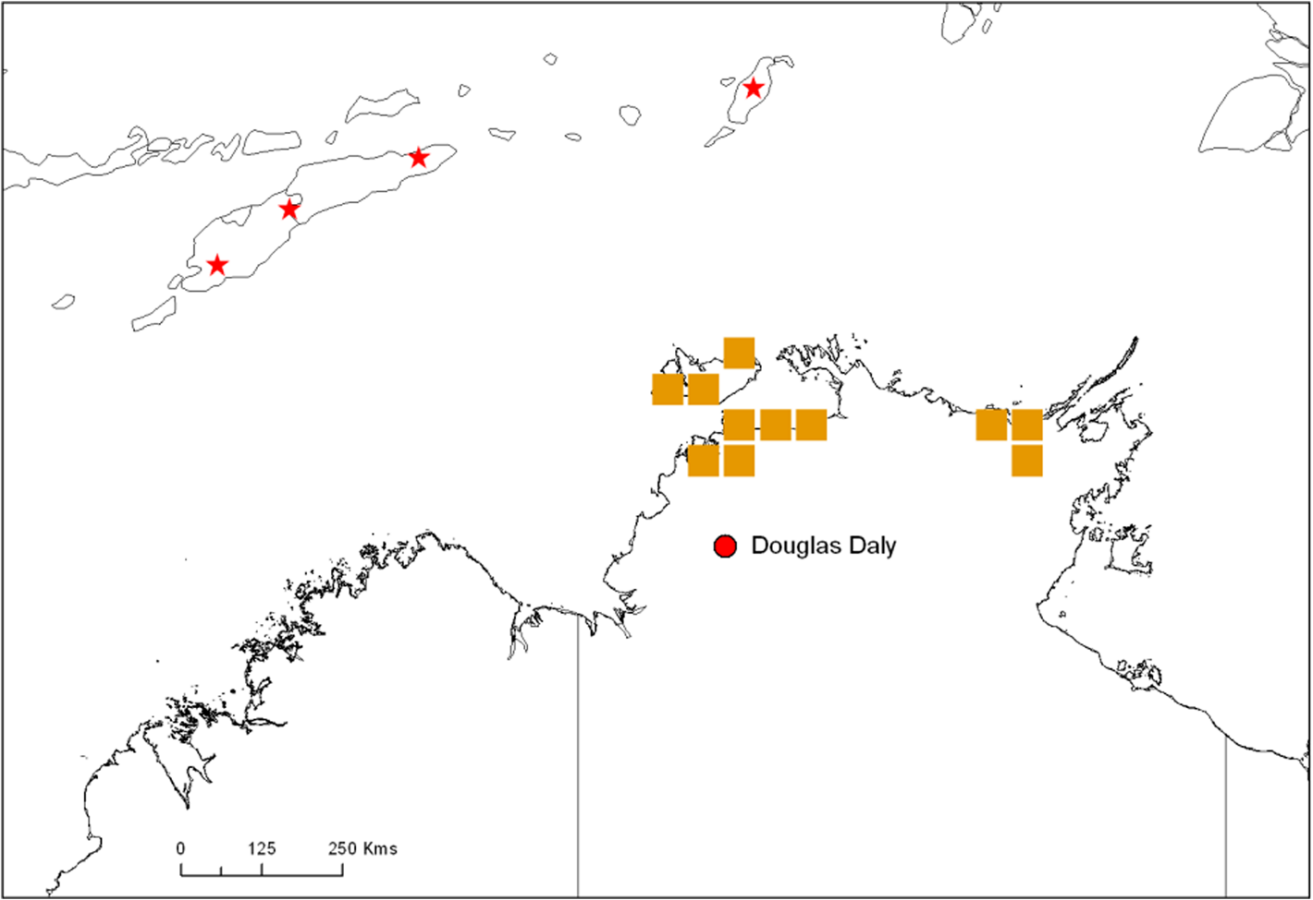
**Dispersal to northern NT in December 2008-February 2009, prior to the collection of two exotic specimens (Case 3) at Douglas Daly (red circle).** The orange squares represent grid cells to which dispersal in the model appears possible during these months; red stars represent the putative source sites.

One female specimen each of *C. palpifer* and *C. flavipunctatus* were collected at Douglas Daly in NT in May 2009 [39]. The previous collection at this site was on 7–8 April 2009. There were no dates within the dispersal window (15 April – 5 May) in which dispersal to the approximate site – or anywhere in NT - appeared possible from Timor-Leste, Indonesia or PNG. Both species (*C. palpifer* and *C. flavipunctatus*) are considered endemic across the region, having been collected across the Indonesian archipelago from West Java to West Papua [40].In order to further explore this case, a number of alternative possibilities were investigated. Firstly the flight duration in the model was increased to 30 hours. Again, no likely dispersal dates or source sites were identified. To allow for the possibility that both collected specimens may have been part of newly established populations, as opposed to the actual dispersed individuals, the dispersal window was extended to include the entire ‘high risk’ period (December-March) prior to the collection, as well as the start of April. Dispersal to the vicinity of the collection site did not appear likely during this period. Dispersal to more northern sites in NT did appear possible in December 2007, January and February 2008 (Figure 4).

### Case 4: *C. orientalis* Macfie, Boigu Is, 24–27 March 2009

A single specimen of *C. orientalis* was collected on Boigu Island in the Torres Strait in March 2009 [39]. The previous collection on at this site was on 23–26 February 2009. This island is in less than 4 km from the PNG mainland.Dispersal from the southern PNG mainland appears to have been possible on a number of occasions within the dispersal window (between the dates of 2–13 and 17–22 March). As with the incursion on Saibai Island, dispersal is likely to have occurred in less than 20 hours (Figure 3B, C).

### Case 5: BTV-2, Beatrice Hill, 10 January 2008

The first BTV-2 detected in Australia was from a sentinel cow at Beatrice Hill on 10 January 2008 [39]. By May 2008, more than half of the sentinel herd were positive for BTV-2. The virus was also detected in samples from cattle at Douglas Daly in the same season.

The modelling indicates that dispersal to the vicinity of the collection site could have occurred one week (3–4 January) prior to sample collection, from a site within West Timor (Figure 3D). Recent full genome analysis of the prototype Australian BTV-2 showed that eight of the ten genome segments were more closely related to Asian than Australian viruses [28], supporting the conclusion that this virus is a recent introduction. Surveys of ruminants for BTV antibodies in Indonesia in the 1990s detected serological reactors to BTV-2, suggesting exposure to this serotype [41] and in the source region.

### Case 6: BTV-7, Beatrice Hill, 15 March 2007

Australia’s first isolate of BTV-7 came from a blood sample collected on 15 March 2007 from a sentinel cow at Beatrice Hill [27]. The previous sample collection from this herd was on 8 March 2007, one week earlier. BTV-7 was subsequently detected in another three of the 24 sentinel cattle between 19 April and 28 June 2007 but was not detected in any other NT sentinel herd in that season [27]. The next detection of BTV-7 was in 2010.

A potential dispersal event occurred approximately two weeks (3–5 March) prior to sample collection (Figure 3F), from sites across West Timor and Timor-Leste, as well as two months (14–16 Jan) prior (Figure 3E), from the same source region. BTV-7 has previously been isolated from cattle in Indonesia [42].

## Discussion

To date, all previous aerial dispersal modelling of *Culicoides* has been in the context of emergency animal disease outbreaks. In this study we have used, for the first time, results of routine (‘active’) arbovirus surveillance to strengthen predictions made by an ADM of *Culicoides* long-distance dispersal in a non-disease context. A likely or possible source and period of incursion was identified for five of the six cases evaluated. In each of these cases, modelling indicated that dispersal could possibly have originated from within the previously predicted source region spanning the Indonesian archipelago east of Lombok across to southern PNG and including the island of Timor. Of these five incursions, all but one was likely to have occurred in either January or March, within the previously described ‘high risk’ period of dispersal [10] for *Culicoides* into Australia. The incursion outside of this period and the case which could not be resolved using the model are discussed below.

The detection of *C. flavipunctatus* on Saibai Island in October is outside of the ‘high risk’ period of December-March proposed by Eagles *et al*. [10]. However, that study assessed dispersal into mainland Australia, approximately 150 km further south of Sabai Island and it is conceivable that dispersal events are both more frequent and across a broader ‘season’ for the short distance between southern PNG and the islands of the Torres Strait. It is likely that secondary dispersal from these islands into mainland Australia is restricted to the previously determined ‘high risk’ period. This distinction may be important if an exotic species of *Culicoides* or novel virus becomes established on one of the islands.

In the case of the specimens collected at Douglas Daly (Case 3), no site of origin from outside Australia (given their exotic status) or date of incursion could be determined. This makes it an important case to explore further in relation to the sensitivity of the surveillance system and the input parameters to the model. The *Culicoides* ‘flight’ duration in this study was set at 20 hours and dispersal to five out of six sites appears possible within this timeframe. However, it has previously been shown that this input parameter is the one to which the model system is most sensitive [10]. Whilst dispersal beyond 20 hours may be possible, the Douglas Daly case was re-evaluated in the model with a 30 hour dispersal duration (results not shown) with still no identification of a likely source site outside of Australia. Based on previous studies [9,10], a dispersal duration of longer than this seems highly unlikely, but this may be an important area for further research. Secondly, consecutive dispersal events where a single insect arrives at one site in Australia then subsequently moves to a different site and enters a trap may be possible and, again, this is an area where future studies could assist with ongoing model validation and confidence in model predictions. A constraint with the model set-up that may have contributed to the inability to identify a dispersal event for this case is the low resolution of the meteorological data, although over the long distances evaluated this is less likely to have an impact.

Additionally, the sensitivity of the light trap network as a surveillance system for exotic *Culicoides* species must be considered, with the implication that the detected specimens may have been part of an already established population. Although the network of traps in northern Australia is extensive (Figure 1) there are a range of factors that can affect the sensitivity of vector surveillance, including trap- and species-specific factors, meteorological conditions at time of trapping and proximity of traps to established *Culicoides* populations [30,43,44]. Interestingly, the Douglas Daly specimens were collected outside of the previously described ‘high risk’ incursion period, but they have not been collected at any site within the NAMP network since their first detection and it appears unlikely either species has established in Australia.

In both cases of novel virus detection presented in this paper a possible incursion date was identified within the ten days prior to sample collection. This time is considered sufficient for a dispersed (infected) vector to find, bite and infect a mammalian host and for the minimum incubation period to be met in that host. It is also concurs with the period between likely dispersal event and onset of clinical signs in a BTV outbreak in Sweden [6]. In the case of BTV-7, a dispersal event may also have occurred two months prior to its detection. Given the highly variable biological nature of BTV viruses [25] including variation in infection rates and virus persistence [32], it is difficult to determine which of the two identified dispersal events was the more likely incursion event.

The question arises, can detections in sentinel herds and vector traps (forms of ‘active’ surveillance) be used equally well for model appraisal as outbreak data (a form of “passive” surveillance), which has been so heavily relied upon previously for assessment of *Culicoides* dispersal? Due to the number of sentinel herds, the frequency of testing, and the use of supplementary sero-surveys, the sensitivity for virus detection is reportedly high [32]. Collection of vectors, by one method only (trapping), is perhaps less sensitive, although it is likely that if a dispersed species becomes established it will ultimately be detected. The sensitivity of passive surveillance, which is reliant on the onset of clinical signs, is also not infallible. Incursion of BTV into a non-endemic zone typically results in infection of susceptible ruminants, but even in these areas infection can occur in the absence of clinical signs of disease. Whilst BTV-8, first detected in Europe in 2006, caused clinical disease in cattle, the presentation of clinical signs in this species is rare, and they are generally considered a silent reservoir for the virus. Even with BTV-8, morbidity in cattle was significantly lower than in sheep, and in infected herds in the Netherlands, only 2.5% of cattle developed clinical signs [45]. Thus, it is possible that the exact date of incursion may not be identified in all cases, dependent on the livestock present at the arrival site, the susceptibility of these to expression of clinical disease and the disease reporting and surveillance mechanisms in place. The importance of this has also previously been noted by Agren [5] and is highlighted in the case of BTV-8 detection in cattle in Norway, based on bulk milk testing rather than clinical signs [6].

**Figure 5:**
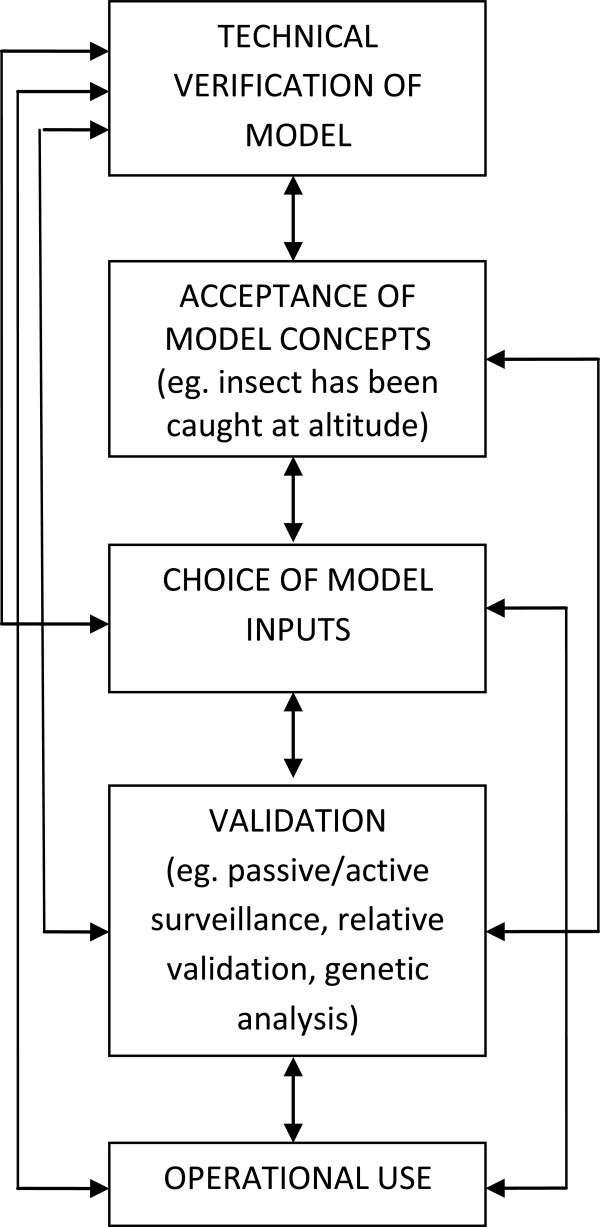
**Steps in model evaluation as they pertain to insect vector dispersal models, adapted from Reeves *et al.*,**[47]**.**

As mentioned in the introduction, rigorous ‘validation’ of these models by use of entomological radars or mark/recapture techniques is currently not feasible. The concept of model evaluation has been extensively discussed and debated in the ecological sciences [46] and has recently been reviewed with respect to epidemiological models in veterinary science [47]. Model evaluation should be considered as following a continuum or spectrum, starting with initial technical verification, followed by acceptance of the model concepts. An example of the latter for dispersal models may include that the insect of interest has been caught at altitude or is able to fly for many hours in laboratory conditions. This is followed by validation, for example by assessment against field data, as shown here, or, as mentioned previously, use of radars or mark/recapture techniques. Validation may also include comparison against other models (‘relative validation’) and sensitivity analysis, which can show to which parameters a model is most sensitive [47]. All these steps lead toward either operational use (as is the aim for many models) and/or the point where a model can be considered “an adequate respresentation of the real system” [46,47] (Figure 5).

Where there is no “conclusive” evidence to validate a model, multiple pieces of information may be combined to support (or otherwise) the model results. With specific respect to insect incursions, Kim [48] proposed a multidisciplinary approach to determining the source of boll weevils collected in an eradication zone. The approach incorporated the results of a model for wind dispersal, along with genetic population analyses of boll weevils caught in the eradication zone and at proposed source sites, and profile analysis of pollen collected on/in the insects [48]. In doing so, they suggested that the application of a multidisciplinary approach increased their confidence in elucidating the source of the incursion.

In two cases presented in this paper – one virus, one vector - genetic analysis of the collected specimen and comparison with conspecific Australian and regional material equivalents [28] (Bellis *et al*., in prep) supports the suggestion that these are (recent) incursions. Clearly, more substantive genetic analysis of both viruses and vectors within the region is necessary to pinpoint the specific sources of *Culicoides* and the viruses they transmit. If such information were available, a similar multidisciplinary approach as used for boll weevils could be applied to *Culicoides* dispersal – combining field data (such as onset of clinical signs in disease outbreaks or collections from sentinel/vector sites), serological status of surrounding herds, analysis of wind dispersal in an ADM and genomic (population genetics and/or specific marker) analysis.

Finally, comparison between incursion detections (both virus and vector) and predictions by the model of the possible frequency of such events are indicative of an “over-prediction” of events by the model. During the period from January 2007-March 2010 there were four detected incursions in total (two virus, two vector), yet in Eagles *et al*.’s [10] previous analysis fourteen possible dispersal events were predicted from the entire source region into Australia. The discrepancy is likely due to a combination of factors, most notably the requirement of more than “just a dispersal event” to occur for successful incursion and detection. Dispersed *Culicoides* must survive long enough on arrival to be caught in a trap or, for successful BTV transmission, to bite a susceptible host. For BTV transmission to occur also requires the vector to be infected at origin – itself dependent on BTV circulation in ruminants at source. Thus, the model framework presented in the previous paper [10], and substantiated here with incursion data, should be considered as indicating the spatial and temporal nature of *dispersal*, and the *relative* frequency of such dispersal from specific source sites, and to arrival regions. As noted above, if the sensitivity of surveillance is not perfect, then some dispersal events predicted by the model – resulting in incursion of vector with or without virus – may not be detected. Additionally, in this paper only novel *serotypes* of BTV and exotic species of *Culicoides* have been investigated. Incursions of BTVs of the same serotype or species of *Culicoides* as those already established in Australia may be expected to occur. Evidence for this comes from the detection of novel *genotypes* of the virus (based on sequencing of segment 3 of the genome) in northern Australia [49]. Subsequent detections of new genotype/serotype combinations may also be incursion events but due to the propensity for genome segment reassortment in BTVs, they cannot be distinguished from reassortments of endemically circulating viruses.

## Conclusions

This study has shown that active surveillance can be used in collaboration with results of an ADM to assess the spatial and temporal nature of *Culicoides* dispersal into an endemic region. Previously, retrospective use of such models has primarily been in response to the presentation of clinically affected animals in outbreaks of disease. Where BTV is already endemic, the concurrent use of an ADM alongside existing BTV and *Culicoides* surveillance may help guide the strategic use of limited surveillance resources as well as contribute to continued model validation and refinement. The same methodology can be applied to other arboviruses with windborne vectors and their relevant surveillance systems.

## Competing interests

The authors declare that they have no competing interests.

## Authors’ contributions

Conceived and designed the experiments: DE, MPZ, PJW, PAD. Conducted modelling: DE. Contributed data for case study: LM, RW, SD, GB. Wrote the paper: DE. All authors contributed to editing, and read and approved the final manuscript.
